# Diagnostic Criteria for Convergence Excess: Diagnostic Validity of Clinical Signs Associated with Near Esophoria

**DOI:** 10.3390/jemr19030053

**Published:** 2026-05-14

**Authors:** Pilar Cacho-Martínez, Mario Cantó-Cerdán, Zaíra Cervera-Sánchez, Ángel García-Muñoz

**Affiliations:** 1 GIOptom—Grupo de Investigación en Optometría, Universidad de Alicante, 03690 Alicante, Spainag.munoz@ua.es (Á.G.-M.); 2 Vissum Grupo Miranza, 03016 Alicante, Spain

**Keywords:** convergence excess, accommodation, ocular, diagnosis, vision, binocular, vision, disorders

## Abstract

To propose which tests may be used for diagnosing convergence excess. A prospective study of a consecutive clinical sample was performed. Patients (18–35 years) attending optometric care underwent subjective refraction, cover test, and Symptom Questionnaire for Visual Dysfunctions (SQVD). Based on cover test and SQVD scores, two groups were recruited: 64 symptomatic subjects with near esophoria and 64 asymptomatic with normal binocular vision. Accommodative and binocular tests were assessed, identifying those with significant statistical differences between groups. Diagnostic validity was analysed using ROC curves, sensitivity, specificity, and likelihood ratios. A serial testing strategy combining tests was also evaluated. ROC analysis showed best diagnostic accuracy for binocular accommodative facility (BAF) failing with −2.00 D (area under the curve, AUC = 0.865) and vergence facility (VF) failing with base-in prisms (AUC = 0.864). Using cutoffs from ROC analysis (BAF: ≤8.25 cpm and VF ≤ 12.75 cpm), their combination showed best validity (S = 0.625, Sp = 0.938, LR+ = 10, LR− = 0.4). The combined AUC was 0.932. The proposal for diagnosing convergence excess is to use, in addition to near esophoria with normal distance heterophoria, the combination of failing BAF with negative lenses and failing vergence facility with base-in prisms.

## 1. Introduction

Convergence excess (CE) is a nonstrabismic binocular disorder in which the subject presents an orthophoria or esophoria at distance vision, with a significant near esophoria, reduced negative fusional vergence (NFV), and a high AC/A ratio [1]. According to the scientific literature, several studies have commonly found this binocular disorder in clinical practice. Thus, a systematic review [2] on the prevalence of general binocular dysfunctions reported that the prevalence rates of CE varied across studies, ranging from 9% to 15%. The discrepancies in prevalence figures were due to both sample population (neither randomised nor representative) and the lack of consistency in diagnostic criteria. Since this systematic review to date, 23 studies have reported the prevalence rates of CE [3,4,5,6,7,8,9,10,11,12,13,14,15,16,17,18,19,20,21,22,23,24,25], ranging from 0.8% to 12.3%. Of all these studies, only four of them [6,8,11,23] have used randomised samples so that most of them have been analysed in clinical samples. As discussed in that systematic review about the knowledge of the prevalence of these anomalies [2], the lack of randomised samples makes it so that the prevalence rates published are not representative of the overall population. In any case, considering data from this systematic review and all the studies published subsequently, it can be established that, to date, the prevalence rates for CE has been encountered ranging from 0.8% to 15% [2,3,4,5,6,7,8,9,10,11,12,13,14,15,16,17,18,19,20,21,22,23,24,25].

According to the diagnostic criteria, as with other binocular dysfunctions, there is no single test that can definitively establish whether a patient has CE. Consequently, clinicians use a battery of signs and symptoms to support its diagnosis [26].

Symptoms are usually associated with near vision tasks, including headache, blurred vision, diplopia, avoid near task, impossibility to maintain clear vision for a reasonable period of time, difficulty in reading, movement of letters, sleeping when reading, asthenopia, eyestrain, tearing [27]. In this sense, considering all the manuscripts about the scientific literature about convergence excess [2,3,4,5,6,7,8,9,10,11,12,13,14,15,16,17,18,19,20,21,22,23,24,25,26,28], most studies include symptoms when diagnosing this anomaly, except several authors [8,12,21,22,29].

According to the signs, in addition to a near esophoria greater than at distance vision, it is possible to find a reduced negative fusional vergence (NFV) at near, reduced vergence facility (VF) with base-in prisms, and binocular accommodative facility (BAF) reduced with −2.00 D, as well as high monocular estimate method (MEM dynamic retinoscopy) finding diminished positive relative accommodation (PRA), near esofixation disparity, near intermittent suppression, and even a limited stereopsis [1,26]. Among all of them, the scientific literature shows that the authors exploring this binocular dysfunction use different clinical signs, with the presence of a near esophoria as the essential clinical sign for diagnosing convergence excess [2,3,4,5,6,7,8,9,10,11,12,13,14,15,16,17,18,19,20,21,22,23,24,25,26,28]. Moreover, even when the same clinical sign is used, differences exist regarding the cutoff values for each optometric test. As an example, considering all manuscripts about convergence excess, the cutoff values for the clinical sign of having a significant near esophoria range from 0.5 Δ to 6 Δ. Thus, the values referenced are ≥0.5 Δ [10], ≥1 Δ [6,14,15,19,24], >2 Δ [4,5,21,23,30,31,32], ≥2 Δ [8,11,22], ≥3 Δ [33], ≥4 Δ [3,9,18,20,25,29], ≥5 Δ [12,17], and ≥6 Δ [34,35], while others only report significant esophoria without a particular value [7] and others do not describe anything about its value [13,16]. Consequently, the same patient could be diagnosed as having the anomaly or not, depending on the criterion selected.

However, the greater difficulty of the studies which analyse the diagnosis of this anomaly is the lack of epidemiological tools to justify the use of several tests, as well as its values of reference. In fact, none of the existing reports about the diagnosis of this condition evaluate the diagnostic validity of the clinical signs using sensitivity [26], specificity, ROC curves, or likelihood ratios, which are the necessary tools to evaluate the clinical signs that should be used for diagnosing a particular condition [36]. The authors diagnose based upon several criteria they consider patients should have but without justifying why certain clinical signs are taken into account and others are not. This absence of a diagnosis based on epidemiologic criteria reveals the need to justify the clinical signs that must be used in the diagnosis of this nonstrabismic binocular anomaly.

For this reason, considering that convergence excess is a nonstrabismic binocular dysfunction associated with a near esophoria, the aim of this study is to identify and propose which tests may be used in addition to near esophoria, for diagnosing convergence excess. Specifically, the aim is to identify which accommodative and binocular tests report anomalous values in subjects with near esophoria and to analyse their diagnostic accuracy by means of ROC analyses and likelihood ratios.

## 2. Materials and Methods

### 2.1. Patients

A prospective study was conducted on a clinical sample from a primary care optometric clinic. Consecutive patients aged 18–35 years seeking optometric care for a variety of clinical reasons underwent a refractive examination. The upper age of 35 years old was established according to other studies to avoid including subjects with pre-presbyopia [10,37]. This research followed the tenets of the Declaration of Helsinki and was approved by the University of Alicante’s Ethics Committee. Informed consent was obtained from all subjects after given the explanation of the nature of the study.

The refractive examination was performed by means of retinoscopy and subjective examination. The subjective examination was carried out using the monocular fogging method with cross-cylinder, followed by binocular balancing to a standard endpoint of maximum plus for best visual acuity. Once the maximum plus value for best visual acuity was obtained, this result of the subjective examination was then used as the baseline for all future accommodative and binocular vision tests.

The cover test was then performed to determine the patient’s binocular status. Distance (6 m) and near vison (40 cm) cover tests were performed with the subject’s subjective refraction in place using a trial frame. A cover–uncover test was first performed to rule out a tropia, and the alternate cover test (ACT) was done to evaluate the heterophoria status. For objective procedure of prism neutralised ACT, the patient was instructed to fixate on a single letter of 20/30 visual acuity. Using a prism bar, the phoria value was midway between the low and high neutral findings using an ACT [38].

Following the ACT, the SQVD [39] was completed by all patients. This validated questionnaire detects visual symptoms related to any type of visual dysfunctions, so it was used to detect the presence and frequency of visual symptomatology not to diagnose visual anomalies. It has 14 items with a Likert scale of three options to indicate the frequency of the symptom: no; occasionally or often; and almost always. The answer for each item is assigned a score between 0 and 2 points, and the total SQVD score is then obtained by adding the 14 individual item scores. The scores range from 0 to 28. Patient scores ≥ 6 indicate the presence of visual symptoms related to any type of visual dysfunction (refractive, accommodative or binocular).

Taking into account ACT results and SQVD scores, patients were divided into two groups: patients with near esophoria and symptoms (ESO group), and the group with normal binocular vision, that is, normal heterophoria at near and distance vision without visual symptoms (named as control group). The inclusion criteria for both groups of subjects are shown in Table 1. According to the signs, as the expected value of near phoria [1,40,41] ranges between orthophoria and 5.5 ∆ of exophoria, the low limit was selected to consider 1 Δ of esophoria as near esophoria [41]. This cutoff is also evidenced-based, as diagnostic accuracy studies should include subjects with different degrees of the condition to represent the full spectrum of the condition and avoid selection bias. Limiting the sample to subjects with larger deviations may lead to an overestimation of diagnostic accuracy [36]. In relation to symptoms, subjects were considered symptomatic when a SQVD score was ≥6.

As the sample was consecutive, participants were examined sequentially as they attended the clinic. Subjects who met the inclusion criteria were enrolled until 128 subjects (19–35 years, mean: 24.09 ± 4.13 years old) were selected, comprising 64 participants with near esophoria and symptoms (ESO group) and 64 participants with normal heterophoria (control group) and without symptoms. This recruitment strategy minimises selection bias and reflects routine clinical practice. Table 2 shows the characteristics of each group of patients according to their ages, the habitual prescription and the subjective refraction both presented with M, J0, and J45, near cover test and SQVD scores. There were no statistically significant differences between the habitual prescription and the subjective refraction for any of the power vectors M, J0, and J45 (*p* > 0.05).

For each group of patients, with the results of the subjective refractive examination in place, all accommodative and binocular tests determining the patient’s accommodative and vergence status were performed [6]. Accommodative tests were: monocular accommodative amplitude (AA) with push-up method; monocular and binocular accommodative facility (MAF, BAF) at 40 cm using ±2.00 D flip lenses and a 20/30 target (Vectogram 9, Bernell, Mishawaka, IN, USA) with suppression control for binocular measurement, in which the patient had to report when the target was clear; MEM dynamic retinoscopy using trial lenses; and positive and negative relative accommodations (PRA, NRA) while the patient was fixating the horizontal line of 20/30 letters at 40 cm. Binocular tests were: negative fusional vergence (NFV) (blur, break and recovery) at 40 cm with an accommodative target of 20/30 visual acuity; vergence facility (VF) at 40 cm (accommodative target of 20/30 VA) using loose prisms with the combination of 3 Δ base-in with 12 Δ base-out; gradient AC/A ratio at near with minus lenses (−1 D); near point of convergence (NPC) with an accommodative target of 20/30 VA (break and recovery); and Worth test and stereopsis with graded circles of Randot^®^ SO-002 test (Stereo Optical Company, Inc., Chicago, IL, USA).

To minimise potential bias, two independent examiners performed the assessments, with one examiner assessing the cover test and SQVD tool and the other, blinded to these results, performing the remaining accommodative and binocular tests.

### 2.2. Statistical Analysis

Using the accommodative and binocular test results from both groups, the U Mann–Whitney test for two independent samples was performed to detect whether statistically significant differences existed between the two groups. For monocular tests, a comparison between the right and left eyes was previously conducted. As no significant differences between both eyes (*p* > 0.05) were observed, only right-eye results were used in the analysis.

For those tests showing statistically significant differences (*p* < 0.05), the diagnostic validity by means of ROC analysis, sensitivity (S), specificity (Sp), and positive and negative likelihood ratios (LR+, LR−) was assessed [36]. This requires the relation between the result of the diagnostic test being analysed (in this case, each accommodative or binocular test) and the presence of the condition (the gold standard), which in this study is the esophoria at near. Accordingly, S is the proportion of patients of ESO group who have a positive test result and Sp is the proportion of people of control group who have a negative test result. Because diagnostic accuracy must be calculated by comparing each index test to the reference standard, participants must be classified according to the gold standard outcome (presence or absence of near esophoria). Consequently, ROC curves and likelihood ratios can only be computed for tests other than the gold standard. In this case, the cover test, used as the gold standard to classify participants, cannot be evaluated for diagnostic accuracy, as a test cannot be validated against itself.

The likelihood ratio (LR) summarises the diagnostic performance of a test [42]. The positive likelihood ratio (LR+) indicates how much the probability of the condition increases after a positive result, whereas the negative likelihood ratio (LR−) indicates the corresponding decrease after a negative result. General guidelines suggest that LR values > 1 indicates an increased probability that the condition is present, and LR < 1 a decreased probability that the condition is present. The further the LR is away from one, towards either zero or infinity, the stronger the evidence provided by the test result. Clinically, LR+ > 10 indicates that the test provides an excellent ability to rule in the condition, 5–10 is good, 2–5 moderate and 1–2 poor ability to rule in the condition. For LR−, values between 0.5 and 1 indicate that the test has poor ability to rule out the condition, 0.2–0.5 fair/moderate ability, 0.1–0.2 good, and <0.1 indicate the test is excellent for ruling out the condition [42,43].

A receiver operator characteristic (ROC) curve plots the true positive rate (S) against the false positive rate (1-Sp) across a range of cutoff values. The best cutoff point is generally considered to lie at or near the “shoulder” of the ROC curve, because as S is progressively increased, there is little or no loss in Sp until very high levels of S are reached. Accordingly, the overall diagnostic accuracy of a test can be represented by the area under the curve (AUC), so that the larger the area, the better the test performance. An AUC value of 1 indicates perfect test accuracy, with both S and Sp equal to 1 [36].

Accordingly, to analyse which tests had the better diagnostic accuracy, for those tests which had obtained significant statistical differences between both groups, ROC areas were examined. For the analysis of diagnostic validity, it was decided to only select those variables with AUC higher than 0.8 [44] as this value is considered good or excellent. For those variables with AUC higher than 0.8, the curve coordinates (the cutoff points for each test) were examined. The choice of these cutoff points was made by means of a balance between S and Sp, in addition to Youden’s index [44]. These cutoff values are necessary to determine the number of patients who pass or fail each test.

Once the diagnostic validity of each test had been evaluated individually, the same analysis was carried out considering multiple tests using a serial testing strategy [36]. In this approach, a diagnosis is considered when all the selected tests in the sequence (those with AUC higher than 0.8) are failed. For that, the order used was from the greater to the lesser accurate test based upon the area under the ROC curve. First of all, it was considered when the subject failed the most accurate test. Secondly, the subject failed the combination of the two tests with better area and so on until all tests with the better ROC areas (AUC higher than 0.8) were taken into account. The selection of the proposal tests for diagnosing CE was obtained according to the best results.

Once the tests with the highest diagnostic validity had been identified, an ROC analysis of the combined tests was performed. First, a binary logistic regression model was fitted, with the presence of near esophoria as the dependent variable and the previously selected tests as independent variables. Regression coefficients, their standard errors, and odds ratios (ORs) with 95% confidence intervals (CIs) were then estimated to derive a linear predictor (*score*) for each participant, defined as the weighted combination of the independent variables according to their regression coefficients. Subsequently, an ROC curve was constructed for the combined model using this *score* as the predictor variable, allowing calculation of the AUC as an overall measure of the multivariable model’s discriminative ability. Statistical comparisons between the AUC of the combined model and AUC of the individual tests were performed using DeLong’s nonparametric test for correlated ROC curves.

All the statistical and epidemiologic analysis was performed using the statistical software SPSS 29.0 for Windows and R version 4.5.3. Sample size estimation was based on diagnostic accuracy analysis using receiver operating characteristic (ROC) curves. Assuming an expected area under the curve (AUC) of at least 0.80 and requiring a marginal error of 0.10 with 95% confidence, a sample of at least 46 subjects is required for each group [45]. Accordingly, the sample size used in this study is highly adequate to the aim of the research.

## 3. Results

Table 3 shows the results of the Mann–Whitney U test for two independent samples, where tests with statistically significant differences (*p* < 0.05) have been highlighted. Results of comparing the right- and left-eye values for MAF and MEM tests showed that there were no significant statistical differences in both cases (*p* > 0.05), so only results of the right eyes were used for the following statistics.

**Figure 1:**
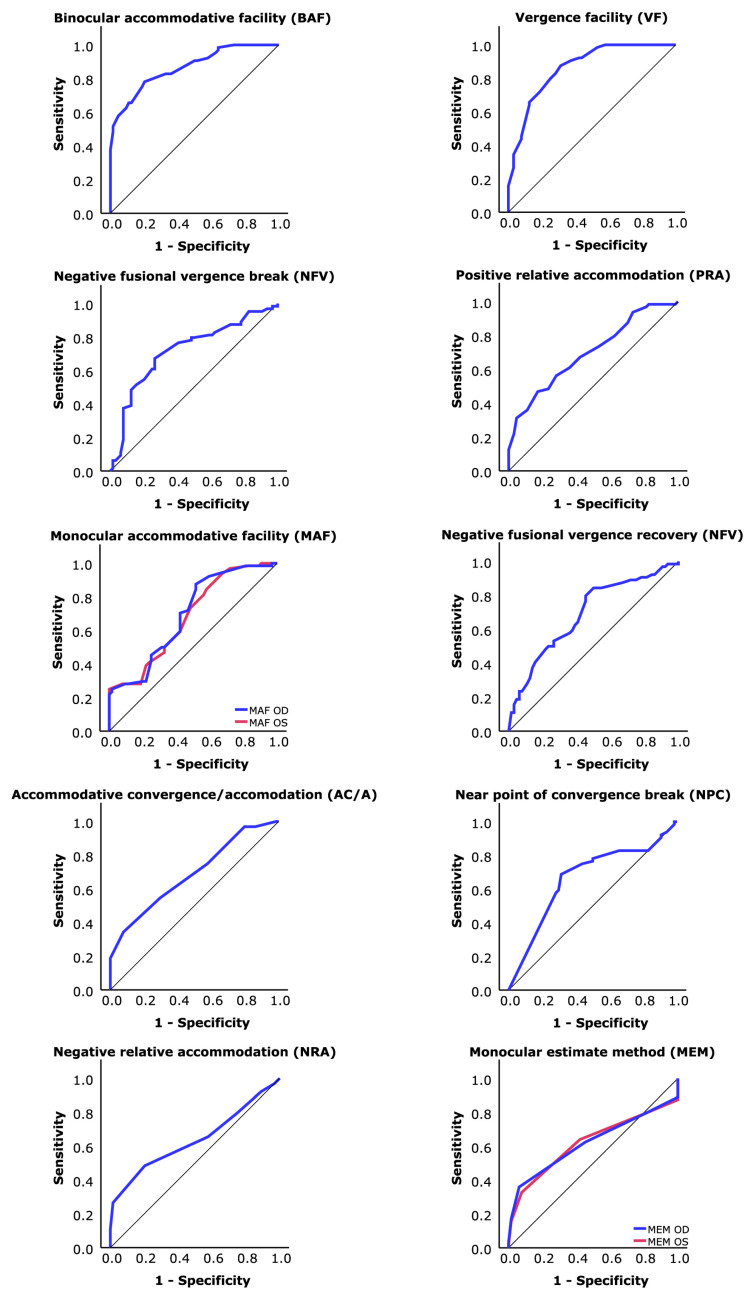
ROC curves for clinical tests with differences between both groups.

Figure 1 reveals ROC curves for each test with statistically significant differences between groups. The areas under ROC curves are showed in Table 4. As only two variables (BAF, VF) had AUC higher than 0.8 [44], the diagnostic validity was only examined for both tests. The selected coordinates (cutoff) for these tests, BAF and VF, are shown in Table 5 with their corresponding S, Sp, and Youden’s index. Using these cutoff points, diagnostic accuracy was obtained for each test. Table 6 shows the results of S, Sp, and likelihood ratios considering each variable alone and both tests as a serial testing strategy.

In the binary logistic regression model, both BAF and VF were included as independent variables. Table 7 presents the regression coefficients of this model, which yielded the following equation: *score* = 7.176 − 0.349 BAF − 0.357 VF. The ROC analysis using this *score* yielded an AUC of 0.932 (95% CI: 0.891, 0.973), z = 20.571, and *p* < 0.001. This combined AUC was significantly greater than the AUC of BAF alone (z = −2.999, *p* = 0.003) and the AUC of VF alone (z = −2.883, *p* = 0.004), according to DeLong’s test. No significant difference was found between the AUC for BAF and VF (z = 0.048, *p* = 0.962).

## 4. Discussion

The results of this research show that in the sample of adults analysed, the tests related to a near esophoria with the highest diagnostic accuracy are the binocular accommodative facility and vergence facility. This supports a proposed evidence-based diagnostic criterion for convergence excess, including the fundamental clinical sign of near esophoria, together with reduced binocular accommodative facility (difficulty focusing with minus lenses) and diminished vergence facility (difficulty fusing with base-in prisms).

The ROC analysis shows that the best AUCs are for binocular accommodative facility and vergence facility, with areas of 0.865 and 0.864, respectively. The ROC analysis shows that the best balance between S, Sp, and Youden’s index is obtained with the cutoff of ≤8.25 cycles per minute (cpm) for BAF and ≤12.75 cpm for VF. Using these cutoffs, diagnostic validity analysis shows that these isolated clinical signs have a good balance between S, Sp, LR+, and LR−.

When considering the combination of tests, the diagnostic validity shows that when both tests are used as serial testing strategy—that is, when the patient fails the BAF with minus lenses (≤8.25 cpm) and the VF with base-in prisms (≤12.75 cpm)—63% of subjects with near esophoria have a positive result (failing both tests). The Sp achieved means that 94% of subjects of control group (without near esophoria) obtained adequate negative results (do not fail both tests). This reduction in S when combining both tests is expected, as serial testing strategies increase Sp at the expense of S. In this context, the aim is not to maximise S for screening purposes but to improve diagnostic confirmation. In terms of diagnostic performance, the positive likelihood ratio (LR+) indicates that subjects with esophoria have a likelihood 10 times greater of having a positive result (BAF and VF failed) compared with the control group. This implies that this combination is an excellent criterion to confirm convergence excess. However, LR− results (0.40) were not so good, indicating that, for the control group, the likelihood of having a negative result (normal BAF and VF) is 2.5 greater than for the esophoria group, which implies that the combination should not be as good as when ruling out the condition. Thus, although BAF and VF individually show good diagnostic performance, their ability to confirm the condition is lower than when used in combination, which supports the use of both tests together in clinical practice. Furthermore, the AUC for the combined BAF and VF (0.932) indicates that this combination is excellent for diagnostic purposes.

Based on these findings, the proposal for diagnosing convergence excess would be to consider a subject who, in addition to near esophoria with normal distance phoria (or a near-distance difference outside a range of 5 ∆), fails BAF testing (≤8.25 cpm) with difficulty in focusing with negative lenses, as well as failing VF (≤12.75 cpm) with difficulty in focusing with base-in prisms. In clinical practice, due to the way of measuring both tests, these cutoffs must be rounded to ≤8 cpm and ≤12.50 cpm for BAF and VF, respectively.

The proposed diagnostic criteria for convergence excess does not coincide with the usually clinical signs used in the scientific literature when analysing not only its diagnosis [26] but also its prevalence [2,3,4,5,6,7,8,9,10,11,12,13,14,15,16,17,18,19,20,21,22,23,24,25] and treatment [28]. Accordingly, the approach of comparing studies is difficult to argue because they are not the same.

The first point of difference is related to the cutoffs used to consider a near esophoria related to convergence excess. Existing studies have used different cutoff values ranging between 0.5 and 6 ∆. Several authors consider that near esophoria must be 0.5 ∆ [10]; others report ≥1 ∆ [6,14,15,19,24]; others consider it to be >2 ∆ [4,5,21,23,30,31,32], ≥2 ∆ [8,11,22], ≥3 ∆ [33], ≥4 ∆ [3,9,18,20,25,33], ≥5 ∆ [12,17], and ≥6 ∆ [34,35]; and there are even several authors who do not specify a value for near esophoria [7,13,16]. In this study, near esophoria has been considered when the patient has the value of 1 Δ or higher. This value has not been arbitrarily chosen but rather determined based on the normative values for heterophoria [1,40,41].

The other point of discrepancy between studies is related to the number of clinical signs used to diagnose this binocular anomaly. In addition to the presence of near esophoria, all scientific evidence on convergence excess since the 1990s has employed a wide range of accommodative and binocular tests [2,3,4,5,6,7,8,9,10,11,12,13,14,15,16,17,18,19,20,21,22,23,24,25,26,28]. It is not possible to designate any single test as more or less prevalent across studies, since most authors chose different combinations of tests drawn from a wide range of accommodative and binocular tests, which do not always coincide even when the total number is similar. In general, in addition to esophoria, the diagnostic battery includes reduced near negative fusional vergence, high AC/A ratio, vergence facility, binocular accommodative facility, MEM, low positive relative accommodation, normal near point of convergence, or esofixation disparity, with reports ranging from one additional clinical sign to more than four. Accordingly, some authors use a total of two clinical signs (esophoria plus one sign) [8,9,12,14,17,20,21,24,25,29,33], whereas others rely on three (esophoria plus two signs) [3,5,6,7,10,11,15,19,22,31], four (esophoria plus three signs) [4,23,30,32], or even five clinical signs (esophoria in addition to four signs) [34]. Several studies do not even describe their diagnostic clinical tests used [13,16].

It is worth noting that among all the studies related to convergence excess [2,3,4,5,6,7,8,9,10,11,12,13,14,15,16,17,18,19,20,21,22,23,24,25,26,28], only some of them incorporate VF as a diagnostic clinical sign [6,10,12,14,17,18,19,22,24,34,35]. The cutoff values used by these authors also vary between studies, since having diplopia with 10 ∆ base-in [34,35], <9 cpm [14], ≤9 cpm [18], <12 cpm [10], ≤12 cpm [12,17], <13 cpm [22], and ≤13 cpm [6,19,24]. In any case, as it can be observed, the values used are near the normative values for VF. As its normative is 16 ± 2.6 cpm [46], the cutoff of <13.4 cpm should be used, so that in clinical settings it is established that the patient fails when the value is ≤13 cpm. As can be observed, the cutoff obtained in this study according to the diagnostic validity analysis (≤12.75 cpm) for VF testing with base-in prisms is similar the normative value established by the scientific literature.

Similar variability exists for BAF testing. Few studies included failure focusing with negative lenses when diagnosing convergence excess [3,4,5,6,9,11,20,22,23,25,30,32], and reported cutoff values range widely: <3 cpm [3,5,6,11,23], ≤3 cpm [4,30,32], <6 cpm [22], and <8 cpm [9,20]. Given a normative value of 8 cpm ± 5 cpm [47], the cutoff <3 cpm should be used to fail the test, which explains why several authors employ values near this cutoff. The use of higher cutoffs (6 [22] and 8 cpm [9,20]) is unjustified, as they are applied arbitrarily without explanation or epidemiological support. Overall, BAT cutoffs reported in the convergence excess scientific literature [3,4,5,6,9,11,20,22,23,25,30,32] differ from that obtained in this study (≤8.25 cpm). Applying the normative cutoff (<3 cpm), the ROC analysis yields a sensitivity of 0.328, a specificity of 1, and Youden’s index of 0.328, indicating inadequate diagnostic performance.

To our knowledge, this is the only study assessing the diagnostic accuracy of all tests related to near esophoria and using diagnostic accuracy to establish optimal BAF cutoffs. Therefore, ROC- and likelihood-ratio-derived cutoffs may differ from traditional normative values. A cutoff of ≤8 cpm with difficulty focusing with negative lenses should be considered a failure related to convergence excess, as it is not arbitrarily defined. Moreover, the good diagnostic validity of BAF suggests that convergence excess may involve alterations in the phasic component of the accommodative controller rather than solely with an insufficient tonic adaptation, as has been reported by several authors [48].

In any case, the differences between the diagnostic criteria for convergence excess proposed in this study (BAF and VF) and those used by other authors are attributable to methodological approaches. The limited research on the diagnosis of this condition has not used epidemiological tools (diagnostic validity of clinical signs) to justify either test selection or their cutoffs [26], and the same applies to prevalence studies [2,3,4,5,6,7,8,9,10,11,12,13,14,15,16,17,18,19,20,21,22,23,24,25]. However, the proposal of this study is justified by an epidemiological analysis and not by the authors’ criteria. Nevertheless, the present study does have limitations. These results can only be attributed to the specific population examined; that is, young adults in a clinical setting without presbyopia. They cannot be extrapolated to the overall population. However, despite this limitation, the main strength of this study is that the recruited patients were drawn from a real-world clinical practice population.

## 5. Conclusions

In summary, this study has shown that, in subjects with near esophoria, the combination of BAF and VF testing has the highest diagnostic accuracy. Accordingly, when a patient presents near esophoria ≥1 Δ (with normal heterophoria at distance or having a difference between both distance and near phoria out of a range of 5 ∆), BAF and VF should be measured. If the patient yields ≤ 8 cpm when failing with minus lenses in BAF testing (±2.00 D) and ≤12.50 cpm when failing with base-in prisms in VF (3∆ base-in/12 ∆ base-out), the clinician should estimate a diagnosis of convergence excess.

The results of this study have important clinical implications. This is the first study about the condition of convergence excess in which epidemiological tools are used to identify which clinical signs are associated with near esophoria. Accordingly, the results of this study add evidence to support the diagnosis of this condition, and this would be helpful for better identifying cases of convergence excess in clinical settings.

## Figures and Tables

**Table 1:** Inclusion criteria for the two groups of participants included in the study.

ESO Group	Control Group
SQVD score ≥ 6.	SQVD score < 6.
Near esophoria ≥ 1 Δ. (As the expected value of near phoria [1,40,41] ranges between orthophoria and 5.5 ∆ of exophoria, the low limit was selected to consider a value of 1Δ of esophoria as a near esophoria) Normative values of distance phoria [40,41] or having a difference between both distance and near phoria out of a range of 5 ∆ [1].	Normative values for distance and near phoria [1,40,41].
Far and near visual acuity ≥20/20 with the best prescription, without ocular motility disorders, vertical deviation, strabismus, or any type of ocular pathology.	Far and near visual acuity ≥20/20 with the best prescription, without ocular motility disorders, vertical deviation, strabismus, or ocular pathology.

**Table 2:** Basal characteristics of each group of patients.

Sample Data		Control Group (N = 64) Average Value ± SD	Esophoria Group (N = 64) Average Value ± SD	Z	*p*-Value
Age		24.09 ± 4.16	24.09 ± 4.12	−0.022	0.983
Habitual prescription					
	M OD	−2.00 ± 2.10	−1.83 ± 2.07	−0.308	0.758
	J0 OD	0.04 ± 0.18	−0.05 ± 0.26	−0.944	0.345
	J45 OD	−0.02 ± 0.10	−0.02 ± 0.13	−0.380	0.704
	M OS	−1.92 ± 2.07	−1.83 ± 2.01	−0.119	0.906
	J0 OS	0.05 ± 0.24	0.01 ± 0.26	−0.416	0.755
	J45 OS	−0.03 ± 0.14	0.01 ± 0.12	−1.478	0.139
Subjective exam					
	M OD	−1.92 ± 2.33	−1.80 ± 2.11	−0.047	0.963
	J0 OD	0.03 ± 0.20	−0.03 ± 0.26	−0.407	0.684
	J45 OD	0.00 ± 0.15	0.00 ± 0.12	−0.431	0.534
	M OS	−1.79 ± 2.23	−1.82 ± 2.00	−0.608	0.543
	J0 OS	0.05 ± 0.26	0.03 ± 0.27	−0.432	0.706
	J45 OS	−0.02 ± 0.14	0.00 ± 0.14	−0.686	0.403
Near cover test		−1.92 ± 1.79	4.83 ± 3.57	−9.802	<0.001 *
SQVD		2.97 ± 1.81	7.61 ± 1.64	−6.588	<0.001 *

* *p* < 0.05 indicates statistically significant differences between both groups.

**Table 3:** Comparison of samples between both groups of patients.

Test	Control Group (N = 64) Average Value ± SD	Esophoria Group (N = 64) Average Value ± SD	Z	*p*-Value
AA OD	11.63 ± 2.23 D	11.51 ± 2.83 D	−0.880	0.379
AA OS	11.64 ± 2.33 D	11.75 ± 3.11 D	−0.393	0.694
MAF OD	13.46 ± 3.55 cpm	10.14 ± 4.83 cpm	−3.802	<0.001 *
MAF OS	13.80 ± 3.90 cpm	10.26 ± 4.92 cpm	−3.545	<0.001 *
BAF	11.81 ± 4.33 cpm	5.20 ± 3.96 cpm	−7.148	<0.001 *
MEM OD	+0.59 ± 0.22 D	+0.71 ± 0.43 D	−2.415	0.016 *
MEM OS	+0.58 ± 0.23 D	+0.70 ± 0.42 D	−2.426	0.015 *
NRA	2.35 ± 0.34 D	2.57 ± 0.49 D	−2.732	0.006 *
PRA	3.59 ± 1.14 D	2.71 ± 1.17 D	−3.870	<0.001 *
AC/A	2.77/1 ± 1.21 ∆/D	3.85/1 ± 1.60 ∆/D	−3.733	<0.001 *
NFV blur	13.68 ± 4.89 ∆	12.31 ± 4.25 ∆	−1.439	0.150
NFV break	19.64 ± 5.54 ∆	15.60 ± 5.99 ∆	−4.283	<0.001 *
NFV recovery	10.57 ± 4.86 ∆	7.39 ± 5.24 ∆	−3.731	<0.001 *
NPC break	3.98 ± 2.14 cm	2.63 ± 2.19 cm	−3.325	0.001 *
NPC recovery	7.23 ± 1.28 cm	7.31 ± 1.58 cm	−0.063	0.950
VF	14.68 ± 3.68 cpm	9.09 ± 3.55 cpm	−7.106	<0.001 *
Stereopsis	38.13 ± 12.92″	44.37 ± 25.01″	−0.981	0.326

AA: accommodative amplitude, MAF: monocular accommodative facility, BAF: binocular accommodative facility, MEM: monocular estimate method, NRA: negative relative accommodation, PRA: positive relative accommodation, AC/A: gradient AC/A ratio, NFV: negative fusional vergence, NPC: near point of convergence, VF: vergence facility, SD: standard deviation, OD: right eye, OS: left eye, D: dioptre, cpm: cycles per minute, ∆: prismatic dioptre, (″): seconds of arch. (* *p* < 0.05 indicates statistically significant differences between both groups).

**Table 4:** Area under the ROC curve for different tests.

Variable	Area	Confidence Interval to 95%		Z	*p*-Value
		Low Limit	Top Limit		
BAF	0.865	0.805	0.926	11.774	<0.001 *
VF	0.864	0.802	0.925	11.742	<0.001 *
NFV break	0.719	0.629	0.810	4.761	<0.001 *
PRA	0.698	0.608	0.788	4.304	<0.001 *
MAF OD	0.694	0.603	0.785	4.217	<0.001 *
NFV recovery	0.691	0.599	0.783	4.064	<0.001 *
AC/A	0.688	0.597	0.778	4.087	<0.001 *
MAF OS	0.681	0.590	0.773	3.851	<0.001 *
NPC break	0.663	0.567	0.760	3.327	0.001 *
NRA	0.637	0.540	0.735	2.740	0.006 *
MEM OD	0.620	0.520	0.720	2.353	0.019 *
MEM OS	0.620	0.520	0.721	2.353	0.018 *

BAF: binocular accommodative facility, VF: vergence facility; NFV: negative fusional vergence; AC/A: accommodative convergence/accommodation; MAF: monocular accommodative facility; PRA: positive relative accommodation; NRA: negative relative accommodation; NPC: near point of convergence, MEM: monocular estimate method, OD: right eye, OS: left eye. (* *p* < 0.05: the obtained area differs statistically from the real value of 0.5).

**Table 5:** Coordinates of each ROC curve.

Variables Turned Out from Contrast	Positive If It Is	Sensitivity	Specificity	Youden’s Index
BAF	≤8.25 cpm	0.750	0.813	0.563
VF	≤12.75 cpm	0.828	0.719	0.547

BAF: binocular accommodative facility; VF: vergence facility.

**Table 6:** Diagnostic validity for tests alone and their combinations using cutoffs derived from ROC analysis.

Test	Cutoff Used	Sensitivity (CI 95%)	Specificity (CI 95%)	LR+ (CI 95%)	LR− (CI 95%)
BAF	BAF ≤ 8.25 cpm	0.750 (0.644–0.856)	0.813 (0.717–0.908)	4.000 (2.356–6.791)	0.308 (0.198–0.478)
VF	FV ≤ 12.75 cpm	0.828 (0.736–0.921)	0.719 (0.619–0.829)	2.944 (1.959–4.425)	0.239 (0.137–0.417)
BAF + VF	BAF ≤ 8.25 cpm FV ≤ 12.75 cpm	0.625 (0.506–0.744)	0.938 (0.878–0.997)	10.000 (3.800–26.318)	0.400 (0.289–0.554)

BAF: binocular accommodative facility, VF: vergence facility, LR+: positive likelihood ratio, LR−: negative likelihood ratio, CI: confidence interval, cpm: cycles per minute.

**Table 7:** Binary logistic regression model for the combination of binocular accommodative facility and vergence facility.

Variable	Coefficient	Standard Error	Wald	*p*-Value	Odds Ratio (95% CI)
BAF	−0.349	0.078	19.794	<0.001	0.706 (0.605, 0.823)
VF	−0.357	0.083	18.459	<0.001	0.700 (0.595, 0.824)
Intercept	7.176	1.263	32.291	<0.001	-

BAF: binocular accommodative facility; VF: vergence facility; CI: confidence interval.

## Data Availability

Data are available upon reasonable request.

## Abbreviations

AA	Accommodative Amplitude
ACT	Alternate Cover Test
AUC	Area Under the Curve
BAF	Binocular Accommodative Facility
CE	Convergence Excess
LR	Likelihood Ratio
NFV	Negative Fusional Vergence
NPC	Near Point of Convergence
PRA	Positive Relative Accommodation
ROC	Receiver Operating Characteristic
SQVD	Symptom Questionnaire for Visual Dysfunctions
VF	Vergence Facility
